# Systematic comparison of biologically active foreign ions-codoped calcium phosphate microparticles on osteogenic differentiation in rat osteoporotic and normal mesenchymal stem cells

**DOI:** 10.18632/oncotarget.16618

**Published:** 2017-03-28

**Authors:** Xiao-Yi Chen, San-Zhong Xu, Xuan-Wei Wang, Xian-Yan Yang, Liang Ma, Lei Zhang, Guo-Jing Yang, Fan Yang, Lin-Hong Wang, Xin-Li Zhang, Kang Ting, Chang-You Gao, Xiao-Zhou Mou, Zhong-Ru Gou, Hai Zou

**Affiliations:** ^1^ Clinical Research Institute, Zhejiang Provincial People's Hospital, Hangzhou 310014, China; ^2^ The First Affiliated Hospital, School of Medicine of Zhejiang University, Hangzhou 310009, China; ^3^ Zhejiang-California International Nanosystems Institute, Zhejiang University, Hangzhou 310058, China; ^4^ Rui'an People's Hospital & The 3rd Affiliated Hospital to Wenzhou Medical University, Rui'an 325005, China; ^5^ Dental and Craniofacial Research Institute, School of Dentistry, University of California, Los Angeles, CA 90095, USA; ^6^ Department of Cardiology, Zhejiang Provincial People's Hospital, Hangzhou 310014, China

**Keywords:** mineral micronutrients, microparticles, osteogenic differentiation, rOMSCs, rMSCs

## Abstract

Osteoporosis is a disease characterized by structural deterioration of bone tissue, leading to skeletal fragility with increased fracture risk. Calcium phosphates (CaPs) are widely used in bone tissue engineering strategies as they have similarities to bone apatite except for the absence of trace elements (TEs) in the CaPs. Bioactive glasses (BGs) have also been used successfully in clinic for craniomaxillofacial and dental applications during the last two decades due to their excellent potential for bonding with bone and inducing osteoblastic differentiation. In this study, we evaluated the osteogenic effects of the ionic dissolution products of the quaternary Si-Sr-Zn-Mg-codoped CaP (TEs-CaP) or 45S5 Bioglass® (45S5 BG), both as mixtures and separately, on rat bone marrow-derived mesenchymal stem cells (rOMSCs & rMSCs) from osteoporotic and normal animals, using an MTT test and Alizarin Red S staining. The materials enhanced cell proliferation and osteogenic differentiation, especially the combination of the BG and TEs-CaP. Analysis by quantitative PCR and ELISA indicated that the expression of osteogenic-specific genes and proteins were elevated. These investigations suggest that the TEs-CaP and 45S5 BG operate synergistically to create an extracellular environment that promotes proliferation and terminal osteogenic differentiation of both osteoporotic and normal rMSCs.

## INTRODUCTION

Osteoporosis is a disease characterized by structural deterioration of bone tissue, leading to skeletal fragility with increased fracture risk. It is estimated that more than 88 million Chinese have osteoporosis and approximately 120 million are at risk of developing the disease. In recent years, numerous techniques and biodegradable materials have been tested to treat bone defects in both postmenopausal women and estrogen depletion-induced osteoporosis in animals [1–5]. Calcium phosphates (CaPs) are widely used in the tissue engineering of bone, including augmentation, replacement and regeneration as they are biocompatible, bioactive and osteoconductive [6–8]. Dynamic and highly vascularized bone tissue can be considered a composite comprising bioceramic (CaP) and biopolymer (mainly collagen) [9]. CaP is the principal mineral component (approximately 69%) of natural bone in the form of carbonated apatite. However, bone apatite is similar to CaP except that bone incorporates trace elements (TEs), for instance, zinc, strontium and silicon. It is reported that strontium can elevate osteogenic differentiation of preosteoblasts and mesenchymal stem cells (MSCs) and *in vivo* it enhances bone formation that prevents osteoporosis [10, 11]. Cardemil et al. have confirmed the favorable osteogenic capacity of Sr-doped CaP (SrCaP) in bone defects in ovariectomised rats [12]. Similarly, Silicon is reported to readily stimulate proliferation and differentiation in osteoblasts and osteoblast-like cells and changes in bone metabolism *in vivo* [13, 14]. Moreover, there is growing evidence that administration of zinc may increase bone formation through stimulation of osteoblast-like cell proliferation and inhibition of osteoclastogenesis and therefore bone resorption [15–17]. On the contrary, zinc-deficiency results in skeletal dysplasia and a significant decrease in bone mineral density (BMD) contributing to osteoporosis [18, 19]. Joshua et al. have evaluated the therapeutic efficacy of Zn-doped tricalcium phosphate (ZnTCP) in treating osteoporosis and found such ZnTCP was effective in preventing and treating bone loss in osteoporotic mice [20]. The extensive bioactive effects of TEs such as Zn^2+^, Sr^2+^, Si^2+^ are currently attracting much interest beyond their nutritional effects in preventing osteoporosis, through incorporation as a constituent of biomaterials [21, 22]. Some individual TE-doped CaP (TE-CaP) has been proven to be more suitable for stimulating bone tissue regeneration in comparison with conventiona pure CaP bioceramics. as demonstrated by our laboratory [23] and others [24].

On the other hand, bioactive glasses (BGs) have also been used successfully in clinic for craniomaxillofacial and dental applications during the last two decades due to their excellent osteogenic potential and ability to bond with bone [25–27]. It is reported that osteogenic cells grow well on BGs *in vitro* and bone matrix production could be improved [28]. Meanwhile, strong bone bonding is observed following implantation of BGs *in vivo*. The first commercially available BG (45SiO_2_-24.5CaO-24.5Na_2_O-6P_2_O_5_; 45S5 Bioglass®), developed by Hench LL. in 1970s, is a melt-derived glass and has been proved to have good osteostimulative and bone-bonding properties [29–31]. Recently, it has been demonstrated that the osteogenic differentiation of rat MSCs is accelerated by exposure to the weak basic ionic dissolution products from 45S5 BG due to culture of the cells on the material [32].

It is well agreed that MSCs are the primary source of osteogenic regeneration [33]. Yu et al. has reported an injectable CaP cement/BG composite with improved properties exhibits promising prospects for bone regeneration [34]. In this study, we hypothesized that the TEs-CaP and 45S5 BG can work synergistically to create an extracellular environment which promotes the proliferation and osteogenic differentiation of MSCs to increase bone formation. We exposed normal rat bone marrow-derived MSCs (rMSCs) and ovariectomized (OVX) osteoporotic rat bone marrow-derived MSCs (rOMSCc) to the ionic dissolution products of the quaternary Si-Sr-Zn-Mg-codoped CaP (TEs-CaP) and 45S5 BG both alone and in combination to compare and evaluate the osteogenic effects of the materials. We investigated cell proliferation and osteogenic differentiation using an MTT test, quantitative PCR, analysis by ELISA and Alizarin Red S staining.

## RESULTS

### Elemental quantification of materials

**Figure 1:**
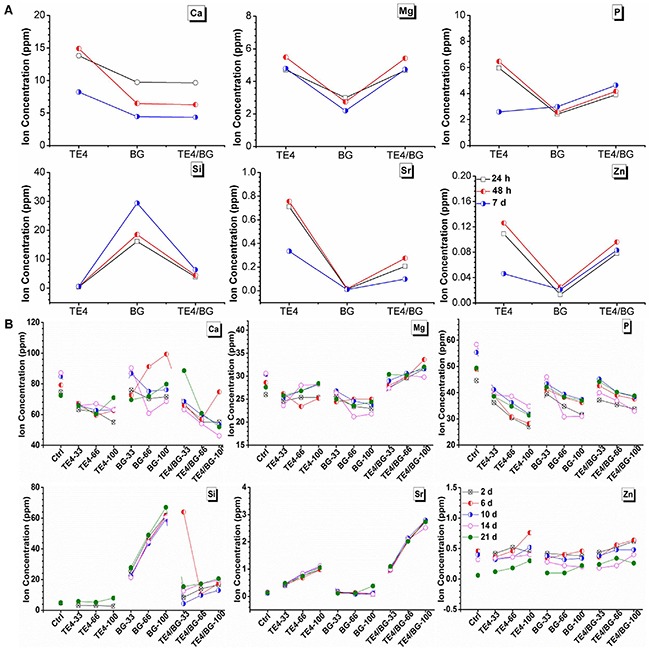
Elemental analysis (ICP-MS) of ion concentration **(A)** Ion concentration in TE4, BG and TE4+BG leachates at various time periods. **(B)** Concentration in conditioned medium of each material group cultured with rOMSCs, changed every two days and collected for ICP analysis.

In the present study, we have conducted ICP-MS elemental analyses of the samples to detect the concentration of each inorganic ion. The ionic concentrations in TE4, BG and TE4+BG leaching liquors at 24 h, 48 h and 7 d are displayed in Figure 1A while that of the culture supernatant of rOMSCs cultured with TE4, BG and TE4+BG displayed in Figure 1B. It should be mentioned the result of rMSCs cultured with TE4, BG and TE4+BG provided results similar to rOMSCs and are thus not shown. It can be seen that the concentrations of zinc and strontium were higher in TE4 and TE4+BG leaching liquor at each time due to the added Sr^2+^ and Zn^2+^ (Figure 1A). When the material leaching liquor was cultured with rOMSCs and rMSCs, the concentrations of Mg^2+^ and Sr^2+^ were higher in the TE4+BG group and Ca^2+^ and Si^2+^ higher in the BG group (Figure 1B). However, the concentrations of P^5+^ and Zn^2+^ were similar in all groups.

### Proliferation of rOMSCs and rMSCs mediated by TE4-CaP and 45S5 bioactive glass

**Figure 2:**
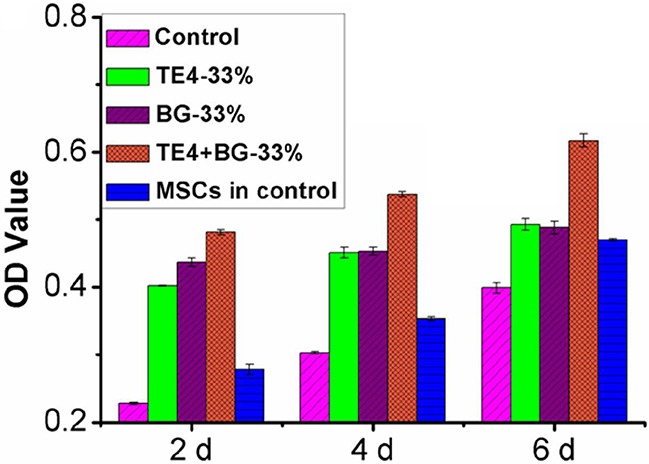
Effect of ionic extraction of materials on cell proliferation activity at the 33% ratio TE4-CaP, BG and TE4-CaP+BG powders acting on rOMSC and rMSC proliferation at 2, 4 and 6 d, measured with MTT test. rOMSCs metabolic activity in TE4, BG and TE4+BG groups were compared with control rMSCs.

The MTT test of metabolic activity indicated the final number of active cells in the conditioned media containing the ionic extracts of TE4, BG and TE4+BG powders (Supplementary Figure 1). Based on this, significantly higher viability of both rOMSCs and rMSCs was observed in the presence of 33% TE4+BG conditioned medium at each time point (*P*<0.05). It should be noted that the viability of rOMSCs in all conditioned media especially the 33% ratio was higher than those of rMSCs in DMEM alone, indicating that the materials elevated the metabolic activity of rOMSCs to equal to that of, or slightly higher than, their normal level (Figure 2). Nevertheless, no significant differences were observed in the other conditions (*P*>0.05).

### ALP activity and mineralization (cell osteogenic differentiation)

**Figure 3:**
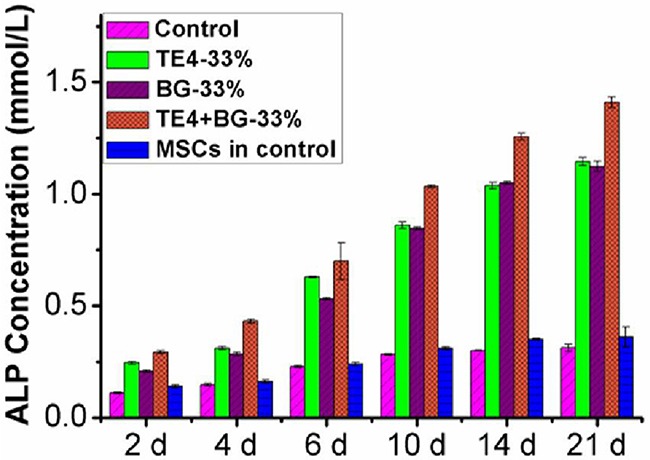
ALP activity in rOMSCs and rMSCs in the different material groups at the 33% ratio rOMSC ALP activity in TE4, BG and TE4+BG groups compared with control rMSCs.

Normalized ALP activity in all groups increased with prolonging culture time, peaking on day 21, as shown in Supplementary Figure 2. Both rOMSCs and rMSCs showed significantly higher ALP activity in the TE4+BG group when compared with the TE4 and BG groups (P<0.05) at every time point, and significantly higher ALP activity in the presence of 33% conditioned medium compared with other volume ratios. When compared with the control group, the TE4 and BG groups were also significantly higher ALP activity but with no significant difference between TE4 and BG groups at each volume ratio. The ALP activity of rOMSCs in all conditioned media, especially the 33% ratio, was higher than that of rMSCs in DMEM only, indicating that the materials elevated the ALP activity of rOMSCs to that of, or slightly greater than, their normal level (Figure 3).

**Figure 4:**
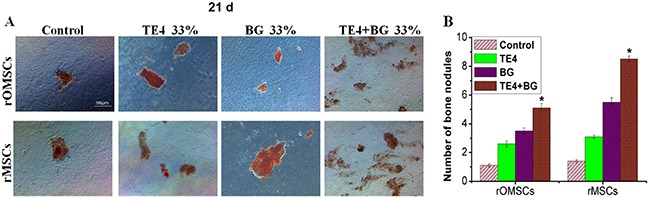
Alizarin Red S staining of bone nodules formed in different groups **(A)** Calcified nodule formation was clearly seen in all groups except cells in control differentiation medium which were lightly stained. **(B)** Number of mineralized nodules in control, TE4, BG, and TE4+BG groups. Mineralized nodule number in the TE4+BG group was significantly higher than that in the BG and TE4 groups (*, *P*<0.05).

Alizarin Red S staining was used to identify calcium deposition in the cultured rOMSCs and rMSCs both in the presence and absence of conditioned media. According to the high magnification views of stained cells (Figure 4A), calcium mineralization was clearly seen in the cells in all groups cultured in the presence of 33% conditioned media, but those cultured in control medium free of particles leaching liquor were only lightly stained. The number of mineralized calcium nodules counted per low-magnification field in rOMSC cultures in the control, TE4, BG and TE4+BG groups was 1.1±0.1, 2.6±0.2, 3.5±0.2 and 5.1±0.3 respectively, and 1.4±0.1, 3.1±0.1, 5.5±0.3 and 8.5±0.2 respectively in the rMSCs cultures. Thus the number of calcium nodules in the TE4+BG groups was significantly higher than that in the TE4 and BG groups (P<0.05, Figure 4B). There was no significant difference between the TE4 and BG groups (P>0.05).

### Expression of specific genes and proteins associated with osteogenesis

**Figure 5:**
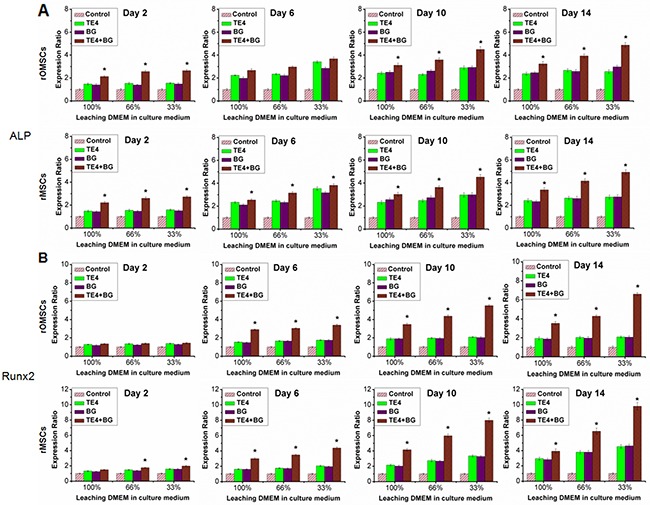
Effect of material powders on early osteogenic differentiation of rOMSCs and rMSCs at the gene level Values were normalized to the control group, set at 1. The TE4-CaP, BG and TE4+BG groups showed elevated expression at each time point. qRT-PCR was used to detect *ALP* **(A)** and *Runx 2* **(B)** expression responsible for osteogenic differentiation, clearly higher in the TE4+BG group than that in TE4 and BG groups (*, P<0.05).

**Figure 6:**
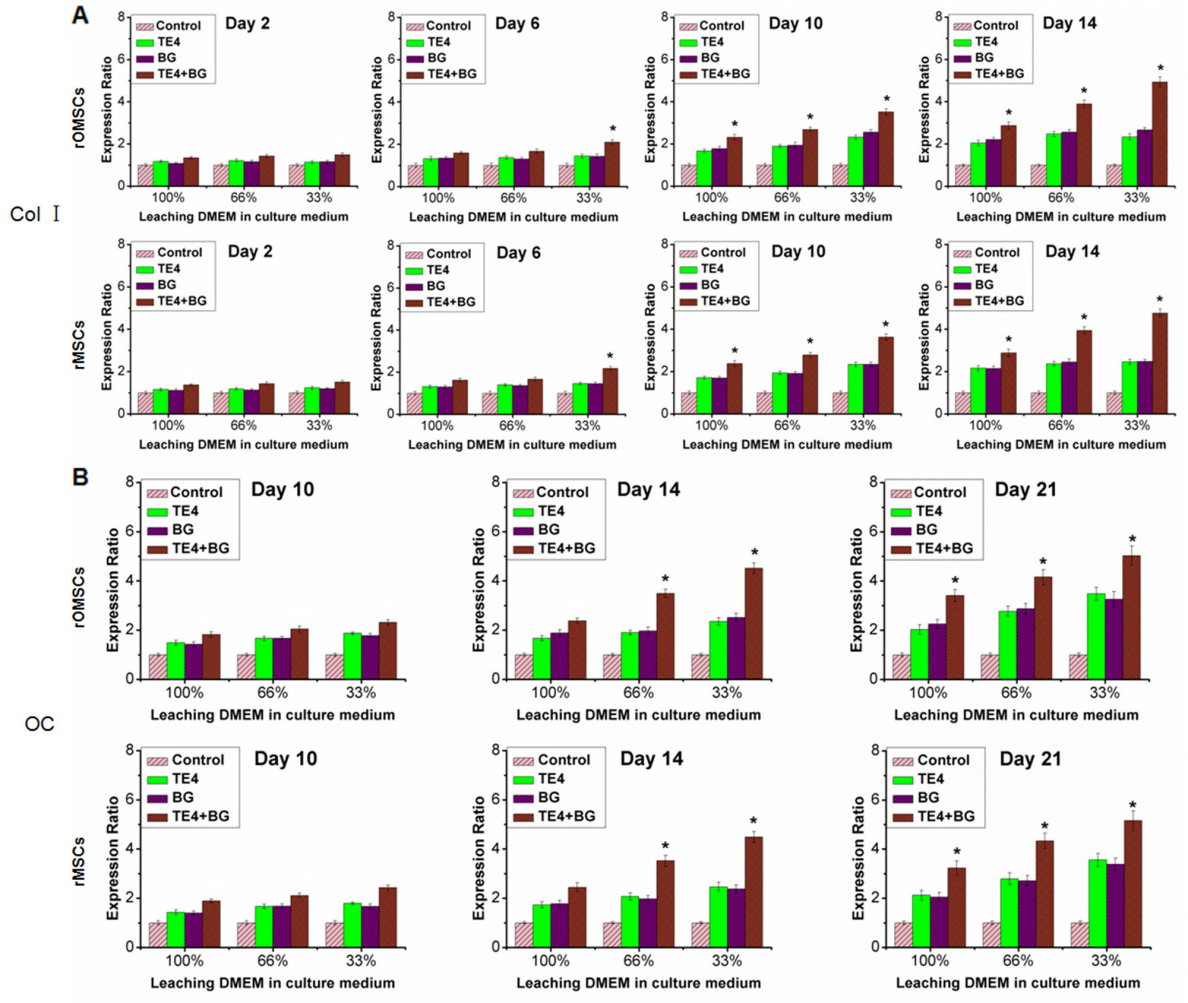
Effect of material powders on later osteogenic differentiation of rOMSCs and rMSCs at the gene level Values were normalized to the control group, set at 1. Expression in the TE4-CaP, BG and TE4+BG groups was elevated compared with the control group at each time point. qRT-PCR was used to detect the *Col I* **(A)** and *OC* **(B)** expression responsible for osteogenic differentiation, clearly higher in the TE4+BG group than that in the TE4-CaP and BG groups (*, P<0.05).

We selected quantitative PCR to detect expression of the genes related to early (i.e. ALP, Runx 2) and late (i.e. Col-I, OC) osteogenic differentiation. ALP expression in both rOMSCs and rMSCs in the TE4+BG group was significantly higher than that of the other groups (P<0.05, Figure 5A) after 2 d of conditioned culture. Runx 2 expression in rOMSCs in the TE4+BG group was clearly higher than that in the other groups at 6 d and longer, while expression in rMSCs in the TE4+BG group was upregulated from 2 d (P<0.05, Figure 5B). Similarly, the expression of Col-I and OC in both cells types increased significantly in the TE4+BG group after 6 and 10 d of conditioned culture respectively (P<0.05, Figure 6A-6B). The expression of all these genes was also higher in the TE4 and BG groups than in the control group but there was no significant difference between the TE4 and BG groups.

**Figure 7:**
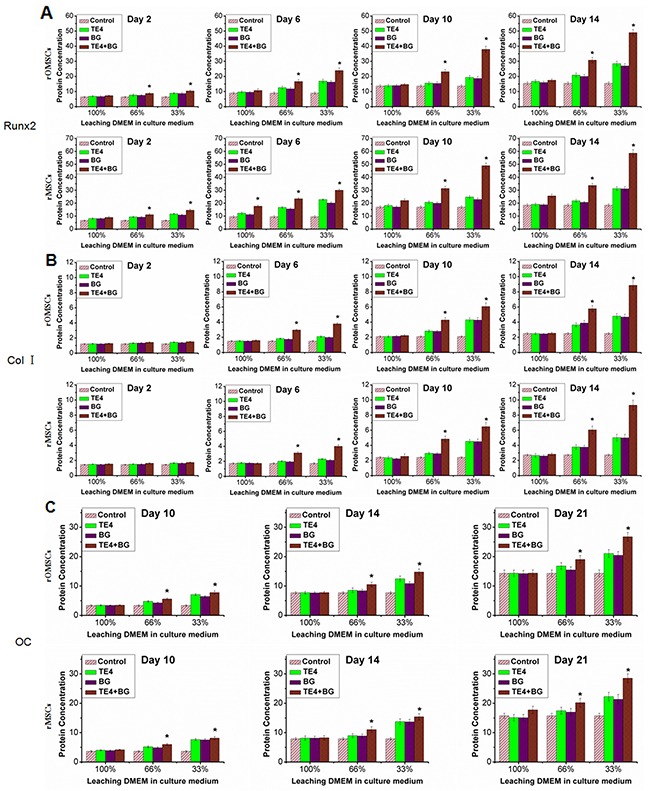
Effect of material powders on osteogenic differentiation of rOMSCs and rMSCs at the protein level Analysis by ELISA was used to detect the Runx 2 **(A)**, Col-I **(B)** and OC **(C)** cytoplasmic protein expression responsible for osteogenic differentiation. Expression in the TE4+BG group was clearly higher than that in the TE4-CaP and BG groups (*, P<0.05).

Concurrent measurement of the cytoplasmic protein concentration indicated early osteogenic differentiation. The expression of Runx 2, Col-I, and OC in both rOMSCs and rMSCs in the TE4+BG group, especially in the presence of 33% conditioned medium, was clearly higher than that in the other groups (P<0.05, Figure 7). Their expression was also higher in the TE4 and BG groups than that in the control group at 33% and 66% volume ratios.

## DISCUSSION

Currently, the search for CaP coatings to improve the osseointegration of implants is preferentially focused on obtaining compositions closer to that of the inorganic mineral phase of bone tissue. Recently, Otsuka et al. evaluated the therapeutic efficacy of CaP bioceramics containing magnesium, zinc and fluoride for improving bone mineral deficiency in OVX rats and found that CaP formulations were effective in improving the composition of bone [24]. This finding was also demonstrated by Tokudome et al. who evaluated the ability of well-characterized Mg/Zn/F-CaP particles to prevent bone mineral deficiency, with results indicating that the compounds were effective in preventing bone loss and having potential use for treating osteoporosis [35]. A complete physicochemical characterization has also been performed on Si/Sr codoped CaP coatings and a significantly positive effect on the osteogenic differentiation of cells was observed, confirming the enormous potential of the approach of such coatings [36].

On the other hand, it is well known that BG has positive effects on bone apposition in both animal studies and clinical trials. The significant effects of 45S5 BG on human bone marrow stromal cell growth and new bone formation were evidenced [37]. An *in vivo* evaluation of porous BGs prepared from 45S5 BG granules indicated that BGs could be a promising material for tissue-engineered bone repair [38]. In clinic, BG particles have been used to contour deficient or irregular craniofacial skeleton on seven patients and so this indicats that BGs could be used clinically as a synthetic bone augmentation clinically [39].

Recently, we have explored a polypeptide-assisted TE dilute doping route to prepare the TEs co-doped octacalcium phosphate (OCP) mesoporous beads with tunable size, mutlilayer structure and controllable TE contents provide close to optimal composition and physicochemical properties for promotion of the early stages of bone regeneration [40]. More recently, we developed a more versatile, facile low-heat biomimetic mineralization route to synthesize a variety of binary to quaternary TE co-doped CaPs in modified SBF. It was found that after injecting the qCaP-loaded hydrogels (pH mediated by minor quantities of commercially available 45S5 BG microparticles) into critical-sized osteoporotic femoral defects *in vivo*, histology and computed tomography scanning revealed that early-stage new bone regeneration was significantly enhanced. The quantity of mature bone substantially increased in rats implanted with qCaP, 12 weeks postoperatively [41]. Therefore, we believe that the combination of TE4 and BG and their superior biological efficacy will provide a breakthrough in the biological repair of pathological bone injuries in the near future.

The aims of this study were to investigate the capacity of the combination of TEs-CaP and 45S5 BG in promoting the proliferation and osteogenic differentiation of MSCs, and especially compare the osteogenic effect of the different materials on pathological and non-pathological rat-derived MSCs. In this regard, it was observed that the proliferation and osteogenic differentiation of both rOMSCs and rMSCs was promoted when cultured in the TE4-conditioned medium in addition to the BG group. Furthermore, the combination of TE4 and BG was found to be more effective than a single material alone. According to the ICP-MS results, the concentration of Mg^2+^ and Sr^2+^ was higher in the TE4+BG group which may indicate that these two ions play a more important role than the others in osteogenic differentiation (Figure 1B). Ossification was also promoted by relatively low concentrations of Si^2+^ and Zn^2+^ or else high concentrations probably lead to some negative effects in bone formation and repairing.

*In vitro* studies have demonstrated that the potential to proliferate and undergo osteoblastic differentiation of MSCs derived from osteoporotic rats and patients is significantly reduced, ultimately causing reduced regeneration capacity compared to that of normal bones [42, 43]. In our study, the metabolism, and thus proliferative potential of rOMSCs was shown to be lower than that of rMSCs in control medium at each time point according to the MTT test, which suggested that the proliferative potential of rOMSCs was decreased compared to rMSCs. Meanwhile, the ALP activity of rOMSCs was also lower than that of rMSCs in control medium indicating that the osteogenicity of rOMSCs was also reduced. However, when exposed to the conditioned culture media of the three groups of materials, the proliferative and the osteoblastic differentiation potential of rOMSCs were significantly elevated and the gap between rOMSCs and rMSCs narrowed especially in the TE4+BG group (Figure 2 and Figure 3).

In this work, we also analyzed the expression of major genes and proteins important for bone formation. Up-regulation of the osteogenic differentiation markers *Runx2*, *Col-I*,*ALP* and *OC* were detected in the TE4+BG groups indicating that TE4+BG induced a distinct osteogenic effect at least at the gene expression level. In fact, the present data demonstrated the similar effect of TE4+BG at the protein expression level of Runx2, Col-I, ALP and OC. On the other hand, our results showed that *Runx 2* expression in rOMSCs in the TE4+BG group was clearly higher than that in the other groups at 6 d and later, while its expression in rMSCs in the TE4+BG group was up-regulated from 2 d (*P*<0.05, Figure 5A). The expression of Runx 2 is essential for cellular commitment to an osteogenic differentiation [44], thus the results suggested that the combination of TE4 and BG is more effective for osteogenic differentiation than either alone. However, at the protein expression level, Runx 2 expression in both rOMSCs and rMSCs in the TE4+BG group was clearly higher than that in the other groups at 2 d and later, suggesting that only a small change in Runx 2 gene expression could cause significantly higher protein expression of Runx2 in rOMSCs than rMSCs. The detailed mechanism by which the materials stimulate MSC proliferation and osteogenic differentiation *in vitro*, and the exact manner by which TE functions in promoting bone formation and repair warrant further investigation.

## CONCLUSIONS

In summary, we exposed osteoporotic and normal rat-derived MSCs to the ion dissolution products of the TEs-CaP and 45S5 BG and a mixture of the two so as to compare and evaluate the osteogenic effects of the different materials. The MTT assay demonstrated that a mixture of TEs-CaP and 45S5 BG can promote the proliferation of MSCs. In addition, analysis of ALP activity and Alizarin Red S staining indicate that the synergistic stimulation of both TEs-CaP and 45S5 BG materials provids a preferential environment for directing rOMSC and rMSC differentiation towards osteoblasts. The gene and protein expression profiles of osteogenic differentiation markers demonstrated that the materials induced rOMSC differentiation through up regulation of Runx2, Col-I, ALP and OC expression, which is similar to the osteogenic stimulation of rMSCs from normal rat hind legs. Our studies demonstrated that the TEs-CaP and 45S5 BG operate together to create an expected extracellular environment that promotes proliferation and the osteogenic differentiation of MSCs to increase bone formation.

## MATERIALS AND METHODS

### Preparation of materials

Reagent-grade inorganic salts (BBI, Canada), Tris (Bio-Rad, USA) and poly-L-aspartic acid (PAsp, Mw 5.5 kDa; Taihe Co., Shandong) were used as received. Deionized water was used in all experiments. Standard simulated body fluid (SBF) containing 142.0 mM Na^+^, 5.0 mM K^+^, 1.5 mM Mg^2+^, 2.5 mM Ca^2+^, 0.5 mM SO_4_^2−^, 1.0 mM HPO_4_^2−^, 147.8 mM Cl^−^, and 4.2 mM HCO_3_^−^ was prepared and buffered to pH 7.4 at 37oC according to Kokubo's recipe. A modified SBF solution containing 20 μM PAsp was prepared with Zn^2+^:Ca^2+^ and Sr^2+^:Ca^2+^ molar ratios of 0.08:2.5 (3.2 at%) and 0.15:2.5 (6.0 at%) respectively. Twenty five mM Na_2_SiO_3_ solution was then added to give an SiO_3_^2−^:HPO_4_^2−^ ratios of 0.08:1.0 (8.0 at%). The pH of the modified SBF was adjusted to 7.4 prior to filtering and processing by hydrothermal treatment at 120oC for 60 min. The TEs-CaP powders (denoted as TE4 group) were harvested by filtering the suspension and drying in a vacuum. The TEs-CaP/BG composite powders (denoted as TE4+BG group) were prepared with 45S5 BG (denoted as BG group) and TEs-CaP powders at a weight ratio of 0.1:1.5 (6.7 wt%).

The elemental composition of the final samples was measured by inductively coupled plasma-mass spectrometry (ICP-MS) (IRIS INTREPID II XSP, Thermo). Sterilized TE4, BG and TE4+BG were respectively immersed in Dulbecco's Modified Eagle Medium (DMEM) at a density of 1.0 mg/ml. The fluid in which ions had leached, termed leaching liquor, was collected at 24 h, 48 h and 7 d after immersion and then diluted using 8% HCl solution for ICP-MS analysis.

### Cell isolation and cultivation

The rOMSCs and rMSCs were obtained from marrow aspirates taken from the femurs of normal and OVX rat hind legs, as described previously [41]. The procedures for the use of animals were in accordance with the animal care and use committee of Zhejiang University. Three-month old female Sprague-Dawley rats of weighting 220±6 g were OVX to induce trabecular bone osteoporosis, a well-accepted technique. rOMSCs were obtained from rats four months after OVX that had been raised on a normal diet,. Briefly, both the OVX and normal rats were sacrificed by deep anesthesia, their femurs harvested and washed with betadine solution and Hank's balanced salt solution containing 2% fetal bovine serum (FBS) and 1% penicillin-streptomycin. All the muscle was cleaned from the bones and the bone marrow flushed out using Hank's buffer. Ultrapure water was added to the marrow to lyze the red blood cells, then the remaining cells spun into a pellet. The supernatant was decanted and the cells were resuspended in culture medium. Cells were then cultured in 25 cm^2^ flasks in DMEM supplemented with 10% FBS and 1% penicillin-streptomycin at 37oC, in 5%CO_2_ within a saturated humidity. Non-adherent cells were removed after 7 days. The culture medium was changed three times per week, and cell experiments performed after the second passage. Once cells had reached confluence they were removed with 0.25% trypsin in 1mM tetrasodium EDTA (Invitrogen, USA). All experiments were performed in accordance with relevant guidelines and regulations of the Zhejiang Provincial People's Hospital. Animal experimentation protocols were approved by the Ethics Committee of the Zhejiang Provincial People's Hospital.

### Preparation of leaching DMEM and conditioned media

The sterilized three groups of powders (i.e. TE4, BG and TE4+BG) were immersed in DMEM at a density of 1.0 mg/ml. The DMEM into which ions had leached, known as leaching liquor, was collected at 48 h after immersion. Conditionized medium comprised the DMEM leaching liquor supplemented with 10% FBS and 1% penicillin-streptomycin. It was divided into three sub-groups by mixing fresh DMEM with conditioned media at volume ratios of 33%, 66% and 100% respectively. The conditioned medium of each material group was cultured with both rOMSCs and rMSCs and was changed every 2 d, the culture supernatant collected for ICP-MS analysis at the time the medium was changed.

### Cell proliferation by MTT test

rOMSCs and rMSCs were plated at a density of 10×10^4^ cells/cm^2^ into 96-well plates and divided into 9 groups. Each group was cultured in a different conditioned medium, fresh DMEM supplemented with 10% FBS and 1% penicillin-streptomycin used as a control. MTT (3-(4,5-dimethyiazolyl-2)-2,5-diphenyl-tetrazolium bromide; Sigma, Germany) is a dye which is reduced into formazan by the metabolic activity of mitochondria in active cells. Cell viability and proliferation rate at 2 d, 4 d and 6 d were measured using an MTT assay. Twenty μl MTT solution (5 mg/ml) were added to each well and the plates incubated (37°C, 5%CO_2_) for 4 h, after which the medium was removed and 100 μl dimethyl sulfoxide (DMSO; Merck, Australia) added. The plates were then covered with tinfoil and placed on an orbital shaker for 10 min. The optical density (OD) at 490 nm was measured by spectrophotometry.

### Alkaline phosphatase (ALP) activity assay

ALP activity was determined at days 2, 4, 6, 10, 14, 21 using a LabAssay™ ALP assay kit (WAKO, Japan). The rOMSCs and rMSCs were washed in phosphate-buffered saline (PBS) three times and lysed with 0.1% Triton X-100 solution. The ALP activity of the lysates was determined using p-nitrophenyl phosphate as a substrate. The absorbance was measured at 405 nm using a microplate reader.

### Gene expression using quantitative PCR analysis

In order to obtain more detailed information about the osteogenic effects of the TE4, BG and TE4+BG powders on rOMSCs and rMSCs, qRT-PCR was used to quantify the upregulation of *runt-related transcription factor 2* (*Runx 2*) and other genes associated with osteogenesis including *alkaline phosphatase* (*ALP*), *type 1 collagen* (*Col-I*) and *osteocalcin* (*OC*). After days 2, 6, 10, 14 and 21, the cell culture medium was removed and total RNA extracted from the cells using the Trizol extraction method. Purified RNA was then used for cDNA synthesis using a PrimeScript™ RT reagent kit (Takara, Dalian) following the manufacturer's instructions. The expression of various transcripts was analyzed using SYBR Premix Ex Taq (Takara, Dalian) and normalized against the house keeping gene *β-actin*. Primer sets used in this study are shown in Table 1.

**Table 1:** Sequence of primers and real-time PCR conditions

Gene	Primer Sequence (Forward/Reverse)	Amplicon Size (bp)	Annealing Temperature (°C)
***β-actin***	5′-TGACCCAGATCATGTTTGAGACCTT-3′	111	62
	5′-CGGAGTCCATCACAATGCCAGT-3′		
***ALP***	5′-CAGGATTGACCACGGGCACC-3′	326	58
	5′-GCCTGGTAGTTGTTGTGAGC-3′		
***Runx 2***	5′-ATTCAGTGACACCACCAGG-3′	469	52
	5′-AAATAGGCATCAGACAAACA-3′		
***Col-I***	5′-TACAGCACGCTTGTGGATG-3′	190	55
	5′-TTGGGATGGAGGGAGTTTA-3′		
***OC***	5′-TAAGGTGGTGAATAGACTCCG-3′	281	54
	5′-GTGCCGTCCATACTTTCG-3′		

### Protein expression using ELISA analysis

rOMSCs and rMSCs were washed in PBS three times and lysed with 0.1% Triton X-100 solution. Cytoplasmic protein expression was measured at days 2, 4, 6, 10, 14 and 21, using a commercially available ELISA kit (Mybiosource, USA) according to the manufacturer's instructions.

### Osteogenic differentiation of cells and alizarin Red S staining

To determine osteogenic differentiation, rOMSCs and rMSCs at second passage were plated at a concentration of 4×10^4^ cells/cm^2^ in four groups: the control group was osteogenic medium only, and three leaching liquor groups containing the leachates of TE4, BG and TE4+BG and osteogenic medium. Osteogenic medium comprised DMEM supplemented with 0.1 μM DMSO, 50 μg.ml^−1^ ascorbic acid, 10 mM glycerol 2-phosphate and 2% FBS.

Mineralization of the cells with the different leaching liquors was used as an indicator of differentiation of the rOMSCs and rMSCs. Following osteogenic differentiation for 21 d, the cell culture medium was removed from each well and the rOMSCs and rMSCs washed in PBS twice prior to fixation in 95% cold ethanol for 30 min at 4oC. Alizarin Red S (Sigma) at a concentration of 0.5% in PBS was added and the cells stained for 20 min at room temperature. Cells were completely washed with PBS and then imaged by light microscopy. The number of bone nodules was counted and compared amongst groups.

### Statistical analysis

Statistical significance was tested using a one-way ANOVA with Bonferroni post-hoc test. All the tests were performed using SPSS with a *P* value <0.05 taken as statistically significant.

## SUPPLEMENTARY FIGURES


