# Supplementary material for: Systematic comparison of biologically active foreign ions-codoped calcium phosphate microparticles on osteogenic differentiation in rat osteoporotic and normal mesenchymal stem cells
Source: Oncotarget. 2017 Mar 28;8(22):36578–90. doi: 10.18632/oncotarget.16618 (PMC5482678; doi:10.18632/oncotarget.16618)
Supplement: Supplementary file 1 [file oncotarget-08-36578-s001.pdf]

Systematic comparison of biologically active foreign ions-codoped calcium phosphate microparticles on osteogenic differentiation in rat osteoporotic and normal mesenchymal stem cells

SUPPLEMENTARY MATERIALS

SUPPLEMENTARY FIGURES

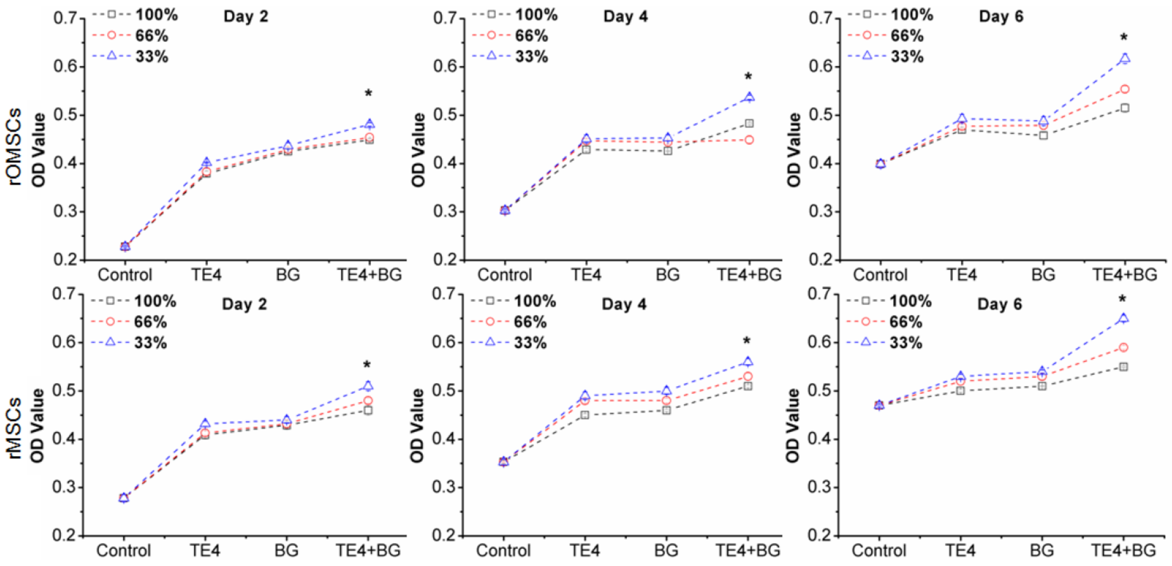

Supplementary Figure 1: Effect of ionic extraction of materials on cell proliferation. TE4+BG group data compared woth TE4, BG and control groups (* p<0.05).

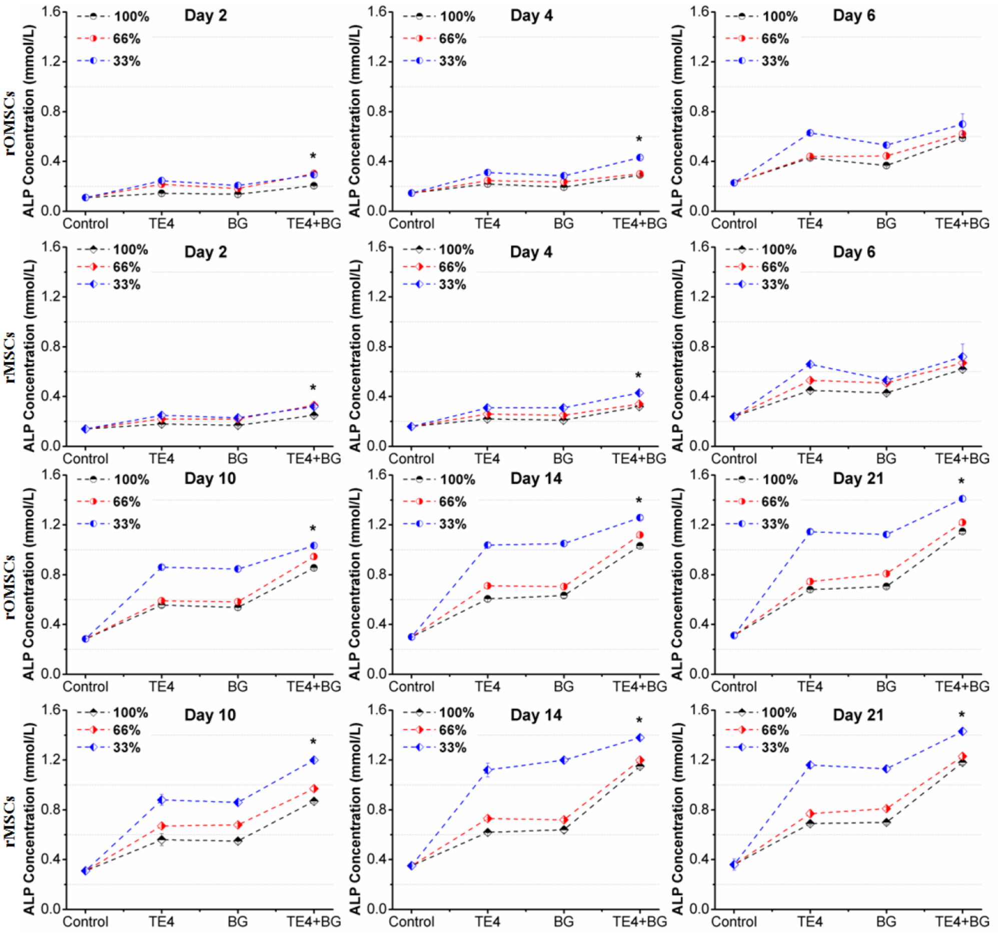

Supplementary Figure 2: ALP activity in rOMSCs and rMSCs in the different material groups. TE4+BG group data compared with TE4, BG and control groups (* $p<0.05$).
